# The relationship between disability and intrinsic capacity among older adults with disabilities in nursing institutions: a cross-sectional study

**DOI:** 10.3389/fpubh.2026.1900440

**Published:** 2026-10-06

**Authors:** Wei Liu, Jiaxin Lv, Zhong Li, Rixin Qin, Kexin Huang, Haiqi Yang, Li Chen

**Affiliations:** 1 School of Nursing, Beihua University, Jilin, China; 2 School of Nursing, Jilin University, Changchun, China

**Keywords:** disability, older adults, healthy aging, intrinsic capacity, long-term care, public health

## Abstract

**Introduction:**

Intrinsic capacity was an important indicator of physical and mental function within the WHO healthy aging framework. Previous studies mainly examined IC decline and its relationship with disability and adverse health outcomes. However, whether IC could reflect functional differences among older adults with disabilities in nursing institutions remained unclear, particularly under the GB/T 42195-2022 Chinese National Disability Classification Standard. This study classified disability degree according to GB/T 42195-2022 and examined whether overall IC and its domains varied across disability degrees. From a public health and long-term care perspective, identifying impaired and preserved capacities could inform health management, reduce care dependence, and support quality of life and healthy aging.

**Methods:**

From August to November 2023, 208 older adults with disabilities were recruited by convenience sampling from five nursing institutions in Jilin Province, China. We collected data through face-to-face interviews, the Integrated Care for Older People tool, the Mini-Mental State Examination, Short Physical Performance Battery, Mini Nutritional Assessment, World Health Organization-recommended visual acuity test, APP-based hearing test, and Patient Health Questionnaire-9. Spearman’s correlation and ordinal logistic regression examined associations.

**Results:**

Intrinsic capacity of older adults with disabilities was impaired across multiple domains. Greater disability degree was associated with more severe IC impairment. However, patterns of variation were not entirely consistent across IC domains and disability degree. The disability degree showed a significant positive correlation with the ICOPE screening score (r = 0.494, *p* < 0.01) and a significant negative correlation with the domain-specific IC measures of motor (r = −0.754, *p* < 0.01), cognitive (r = −0.513, *p* < 0.01), and vitality functions (r = −0.338, *p* < 0.01). The correlations were lower for psychological (r = 0.055, *p* > 0.05), hearing (r = −0.134, *p* > 0.05), and vision functions (r = 0.100, *p* > 0.05).

**Discussion:**

Even after disability occurred, IC could still reflect functional status, while different domains showed distinct patterns across disability degrees. Domain-specific assessment could support targeted care and functional maintenance. In long-term care facilities, care and environmental factors could also influence function. Identifying impaired and preserved IC domains could help reduce care dependence and support quality of life and healthy aging.

## 1 Introduction

Disability referred to a patient’s self-reported difficulty or inability to perform activities of daily living (ADL) or instrumental activities of daily living (IADL) (1). However, in this study, disability degree was defined according to GB/T 42195-2022, *Specification for ability assessment of older adults*, which used a multidimensional assessment rather than a single ADL/IADL measure. Disability was associated with future falls, cognitive decline, hospitalization, and mortality (2). With the global population aging, disability was a major challenge to public health and social care worldwide in the 21st century. Global Burden of Disease (GBD) studies showed that disability caused a large and growing share of health loss worldwide (3). Many older adults still lived with disability, even as people lived longer (4). At the population level, these growing care needs also placed increasing pressure on health and social care systems. If not controlled, disability significantly increased the risk of readmission and frailty degree, reduced quality of life, and even led to death (5–7). In this context, family caregiving capacity was relatively inadequate. More older adults with disabilities were moving to nursing institutions for regular care and health management. Therefore, disability not only affected individual health but also increased care dependence and the burden on nursing institutions.

The World Health Organization emphasized that the fundamental approach to promoting healthy aging was enhancing and maintaining the older adults’ intrinsic capacity (IC). IC referred to total physiological and psychological functions and was mainly divided into five core domains: motor, cognitive, sensory (including vision and hearing), psychological, and vitality functions (8). Research showed that IC decline caused falls, frailty, and readmissions, further affecting older adults’ daily life and increasing the burden on families (9, 10). Research evidence also indicated that IC was an effective predictor of dependence on subsequent care and adverse health events in later life (11, 12). Therefore, older adults identification of IC decline was important for recognizing functional risks in older adults. In the healthy aging framework, IC reflected overall functional health and was related to disability, care dependence, and other adverse health outcomes. Existing IC research mainly focused on early health assessment in older adults and whether IC decline predicted disability, frailty, and other adverse outcomes. A study found that people with lower ICOPE screening scores were more likely to develop ADL disability during follow-up (13). Clearly, the potential link between IC and disability was receiving increasing attention. For older adults with disabilities in nursing institutions, disability degree alone was not enough for ongoing health management. It was also important to identify which functions could be maintained and which might continue to decline. This could provide a basis for risk assessment, resource allocation, and long-term care.

Regarding the impact of IC on disability, existing studies indicated: Reduced intrinsic capacity was linked to a higher risk of disability in older adults. Zhou et al. suggested that older adults with decline in one or more domains of IC had a higher risk of disability than those with no decline (14). Multiple meta-analyses also showed that IC decline was a strong predictor of disability in older adults and highlighted that maintaining higher IC levels reduced disability risk (15, 16). Stolz et al. (13) provided specific correlation data: a 1-point decline in ICOPE screening score increased the risk of ADL disability by 7%, nursing institutions admission by 6%, and mortality by 5%. Review studies also supported the link between older adults’ IC and disability (17). At the functional outcome level, Sánchez et al. indicated that maintaining or improving IC seemed negatively correlated with IADL dysfunction (18). In specific domains, Zeng et al. found that older adults with higher ICOPE screening scores had lower disability risk (19). In addition, declines in cognitive and motor function were linked to higher disability risk one year after discharge. Yu et al. also showed a significant link between cognitive impairment level and IADL obstacles at three years (20). In addition, higher cognitive function was linked to lower disability risk (21). Moreover, vitality was mainly linked to disability through the mediating role of other IC domains. Previous studies showed that IC decline was closely associated with disability and adverse health outcomes. These associations suggested that IC was relevant not only to individual health assessment but also to public health efforts aimed at reducing functional decline and care dependence in aging populations. However, evidence remained limited on whether IC could reflect residual functional capacity and disability degree among older adults with disabilities. However, most studies focused on whether IC decline predicted disability. After disability occurred, less was known about which IC domains were still preserved and which were more closely related to disability severity. It was also unclear whether these differences could guide functional maintenance and individualized care in nursing institutions.

This study aimed to classify older adults with disabilities in nursing institutions according to GB/T 42195-2022. It compared overall IC and its domains across disability degrees. It examined whether the domains changed in the same way. It also analyzed the association between IC and disability degree and evaluated the value of IC at different disability degrees. These analyses could provide a basis for health management and long-term care in nursing institutions, with the goal of maintaining function and supporting healthy aging.

## 2 Materials and methods

### 2.1 Participants and study design

This study was conducted from August to November 2023 in five urban private nursing institutions in Changchun and Jilin, Jilin Province, China. The five institutions were generally similar in operational scale and service characteristics. The study used convenience sampling, and the sample consisted of older adults with disabilities. Inclusion criteria for the study participants were established as follows: (1) ≥ 60 years old; (2) living in nursing institutions; (3) meeting the Ministry of Civil Affairs’ National Standard: Specification for ability assessment of older adults; (4) being fully informed and voluntarily consenting to participate. Exclusion criteria were established as follows: (1) Participants with severe mental illness or cognitive impairment who were unable to understand research instructions or complete the relevant assessments; (2) Participants with severe hearing or visual impairment who were unable to complete the hearing, vision, or other assessments. Used Kendall’s method to estimate the sample size. The sample size was set at 5–10 times the number of variables. This study Used five times the number of variables and adding 20% for invalid questionnaires and missing data, the minimum sample size was 102. The final sample included 208 participants and met the sample size requirement. The present research project was approved by the Ethics Committee of the School of Nursing, Jilin University (ethics number: 2022083103).

### 2.2 Data collection

Data were collected from August to November 2023 through face-to-face interviews. Before data collection, researchers explained the study purpose, content, voluntary participation, and data privacy. Participants gave informed consent and completed the questionnaire. People without consent did not participate. Disability degree came from the assessment records at admission. Each institution had two assessors. They assessed disability independently according to GB/T 42195-2022. If they disagreed, they discussed the case and agreed on the final level. Only the final disability degree was recorded. IC assessments were conducted by two trained researchers with master’s or doctoral degrees in nursing. They did not conduct the disability assessment at admission and did not know the participants’ disability degree.

### 2.3 Instruments

#### 2.3.1 Disability assessment

Disability assessment used the national standard GB/T 42195-2022, Specification for ability assessment of older adults. It contains 26 items in four domains: self-care ability, basic motor ability, mental status, and sensory perception and social participation. The total score ranges from 0 to 90, with higher scores indicating less severe disability. Older adults were classified as level 0 (intact ability), level 1 (mild disability), level 2 (moderate disability), level 3 (severe disability), or level 4 (complete disability). The classification and adjustment rules were shown in Supplementary Table S1. This study included only older adults with disabilities, so levels 1–4 were analyzed. In the ordinal logistic regression, disability degree 1–4 were used as the ordered outcome. A higher level indicated more severe disability. The standard has been validated in nearly 20,000 older adults in China, supporting its reliability and validity.

#### 2.3.2 ICOPE screening tool

We first used GB/T 42195-2022 to identify older adults with disabilities. Then used the WHO-recommended ICOPE screening tool to screen intrinsic capacity. ICOPE screening tools ranged from 0 to 9. A score ≥1 indicated IC impairment, and higher scores indicated more impaired items. This score did not represent a validated overall measure of IC. Further used MMSE, SPPB, MNA, vision, hearing, and PHQ-9 to assess each IC domain. It included six key IC domains: cognitive decline, limited mobility, malnutrition, impaired vision, hearing loss, and depression. ① Cognitive decline: The test included a memory vocabulary test (three words: flower, door, rice), a time and place orientation test, and a short-term memory test. A wrong answer or not recalling any words was a positive screening result. Each item was worth 1 point, totaling 2 points. ② Limited mobility (motor function): The chair stand test observed whether older adults could complete five chair stands within 14 s without using arm strength. If it could not be completed, it was a positive screening result and received 1 point. ③ Malnutrition: Specific tests included: whether there was weight loss of over 3 kilograms in the past three months; whether participants often felt food tasted bland or was hard to swallow. A “yes” response was a positive screening result. Each item was worth 1 point, totaling 2 points. ④ Impaired vision: This was assessed by asking whether participants had visual difficulties, eye disease, or ongoing treatments (e.g., diabetes, hypertension). If present, it was a positive screening result and received 1 point. ⑤ Hearing loss: If the whisper test failed or there was a significant decline in subjective hearing perception during questioning, it was a positive screening result and received 1 point. ⑥ Depression: This was assessed by asking whether participants had felt depressed, discouraged, hopeless, or lacked interest or pleasure in activities in the past two weeks. Any of these was a positive screening result. Each item was worth 1 point, totaling 2 points.

#### 2.3.3 Cognitive function

Used the Mini-Mental State Examination (MMSE) to assess. Folstein et al. created this scale (22). The scale had 30 items, divided into five sections: orientation (10 items), memory (3 items), attention and calculation (5 items), recall (3 items), and language ability (9 items). Each correct answer was worth 1 point, for a total of 30 points. Studies suggested grouping older adults by their educational level and setting corresponding cutoff points. Specifically, for the illiterate group, ≤19 points was the cutoff for cognitive impairment. For those with ≤6 years of education, the cutoff was ≤22 points. For those with a junior high school education or higher, the cutoff was ≤26 points. A score of 24 or higher indicated normal cognitive function based on the original test results. A higher score indicated better cognitive function. The MMSE had good reliability and validity (23), with a Cronbach’s alpha coefficient of 0.833.

#### 2.3.4 Motor function

Used the Short Physical Performance Battery (SPPB) to assess. Yasuda et al. created this scale (24). The scale included three components: standing balance tests, gait test and chair stand test. Each component was scored from 0 to 4, for a total of 12 points. Based on the established scoring criteria, a score of ≥10 indicated normal motor function, while a score of <10 indicated abnormal motor function. A higher score indicated better motor function. The reliability and validity of the SPPB were 0.887, with a Cronbach’s alpha coefficient of 0.868.

#### 2.3.5 Vitality function

Used the Mini Nutritional Assessment (MNA) to assess nutritional status as an indicator of vitality function. Guigoz et al. created this scale (25). The scale included four components: anthropometry, general status, dietary habits, and self-perceived health and nutrition states. The scale had 18 items, with items 1 to 6 evaluated first. A nutritional score > 12 indicated normal nutritional status, and did not need further assessment. If the score was ≤12, it indicated a potential risk of malnutrition, and participants should continue to complete the entire scale assessment. After completing the full assessment, the MNA had a total of 30 points. A score ≥24 indicated good nutritional status; a score between 17 and 24 indicated a risk of malnutrition; and a score <17 confirmed malnutrition. A higher score indicated better vitality function. The MNA had good reliability and validity (26), with a Cronbach’s alpha coefficient of 0.711.

#### 2.3.6 Vision function

Followed the World Health Organization-recommended visual acuity test to measure uncorrected distance and near vision separately. Distance Vision: The older adults removed their eyeglasses and stood 3 m from the chart light box. Measured the left eye, right eye, and both eyes. Used the WHO-recommended international standard vision chart with 6/18 as the threshold for vision impairment. Based on the 3-meter reading method in the “National Standard of the People’s Republic of China (GB11533-2011)”, One-eye vision <4.5 was considered distance vision impairment. Near vision: The older adults removed eyeglasses and stood 33 cm from the standard international near eye chart (GB11533-89). Measured the left eye, right eye, and both eyes and recorded the result in decimal notation. One-eye vision <1.0 was considered near vision impairment. Comprehensive vision assessment: if there was any impairment in distance or near vision acuity for the left eye, right eye, or both eyes, the subject was defined as having abnormal vision function. A higher score indicated better vision function. If all results were normal, the subject was defined as having normal vision.

#### 2.3.7 Hearing function

Used the Tinglibao APP pure-tone test following Luo et al. to assess. The test took place in a quiet room with noise <40 dB. We used the same Tinglibao APP-T100S headset combination for all participants and did not perform extra on-site acoustic calibration. Both ears were tested at 125, 250, 500, 1,000, 2,000, 4,000, and 8,000 Hz. The test started with the better ear, using 1,000 Hz at 70 dB HL as the initial test tone. If the tone was not heard, the intensity was increased by 5 dB. Once heard, the level was decreased by 10 dB and then increased again by 5 dB until the tone was heard again. This process was repeated three times to determine the hearing threshold. PTA4 was calculated separately for each ear as the mean hearing threshold at 500, 1,000, 2,000, and 4,000 Hz. Lower PTA4 indicated better hearing. PTA4 ≤ 35 dB HL indicated normal hearing in one ear. Overall hearing was normal when both ears had PTA4 ≤ 35 dB HL. PTA4 > 35 dB HL in either ear indicated hearing impairment. A previous study found no significant difference between the APP-T100S and GSI-61 pure-tone audiometer under the same test conditions.

#### 2.3.8 Psychological function

Used the Patient Health Questionnaire-9 (PHQ-9) to assess depressive symptoms as an indicator of psychological function. Kroenke et al. created this scale (27). The scale had 9 items. Each component was scored from 0 to 3, for a total of 27 points. Scores of 5, 10, 15, and 20 indicated mild, moderate, moderately severe, and severe depressive symptoms, respectively. Higher scores indicated more severe depressive symptoms. A higher score indicated worse psychological function. The PHQ-9 had good reliability and validity (28), with a Cronbach’s alpha coefficient of 0.8325.

### 2.4 Statistical analyses

This study used IBM SPSS Statistics 26.0 for analysis. Descriptive statistics were performed on the general demographic characteristics, disability degree, and ICOPE screening score and domain-specific IC measures (motor, cognitive, vitality, psychological, hearing, and vision functions) of older adults with disabilities. We assessed the normality of measurement data using the Kolmogorov-Smirnov (K-S) test. Normally distributed data were shown as mean ± standard deviation (SD), while non-normally distributed data were shown as median [interquartile range] (IQR). Technical data were reported as frequency (*n*) and percentage (%). We used Spearman’s correlation analysis to assess the associations between disability degree and the ICOPE screening score and domain-specific IC measures. We further used ordinal logistic regression to examine the associations between domain-specific IC measures and disability degree.

## 3 Results

The questionnaire was informed consent and confidentiality. We distributed and collected a total of 210 questionnaires (100.0% return rate). Two were excluded due to incomplete items, leaving 208 valid questionnaires (99% validity rate).

The study included 208 older adults with disabilities, with an average age of 76.02 ± 10.84. Among them, 108 were male (51.9%) and 100 were female (48.1%). Most of them were of Han ethnicity (96.2%). Most of the older adults had a low education level, with only 7.7% holding an associate degree or higher. A total of 40.4% of the older adults were married. Most older adults worked in manual labor before retirement (60.1%), and more than two-thirds had chronic diseases (76.9%). The study showed that most older adults with disabilities were completely disabled (33.7%), next were mild disability (25.9%), moderate disability (23.6%), and severe disability (16.8%) (Table 1).

**Table 1:** Characteristics of the sample (*n* = 208).

Characteristics	Total sample
Age (mean ± SD)	76.02 ± 10.84
Sex *n* (%)	
Male	108 (51.9)
Female	100 (48.1)
Ethnicity *n* (%)	
Han	200 (96.2)
Ethnic minorities	8 (3.8)
Education level *n* (%)	
Primary school or below	101 (48.5)
Middle school	42 (20.2)
High school or vocational school	49 (23.6)
College degree or above	16 (7.7)
Marital status *n* (%)	
Married	84 (40.4)
Never married	56 (26.9)
Divorced	9 (4.3)
Widowed	59 (28.4)
Occupation before retirement *n* (%)	
Manual labor	125 (60.1)
Intellectual labor	83 (39.9)
Pension *n* (%)	
≤3,000 CNY/month	125 (60.1)
>3,000 CNY/month	83 (39.9)
Chronic diseases *n* (%)	
No	48 (23.1)
Yes	160 (76.9)
Disability degree *n* (%)	
Mild disability	54 (25.9)
Moderate disability	49 (23.6)
Severe disability	35 (16.8)
Complete disability	70 (33.7)

This study used the WHO-recommended ICOPE screening tool to assess the IC of 208 older adults with disabilities. The study divided the older adults into four disability degrees: mild (*n* = 54, 25.9%), moderate (*n* = 49, 23.6%), severe (*n* = 35, 16.8%), and complete disability (*n* = 70, 33.7%). The results showed significant differences in ICOPE screening scores significant differences in ICOPE screening scores at different disability degrees (*F* = 23.025, *p* < 0.001). The mean ICOPE screening score generally increased with disability degree: mild (2.24 ± 1.84), moderate (3.49 ± 1.98), severe (4.80 ± 2.22), and complete disability (4.30 ± 1.29). The score was slightly lower in the complete-disability group than in the severe-disability group. Therefore, the overall pattern was not strictly monotonic (Table 2; Figure 1).

**Table 2:** Intrinsic capacity status of older adults at different disability degrees (*n* = 208).

Variable	Mild disability (*n* = 54)	Moderate disability (*n* = 49)	Severe disability (*n* = 35)	Complete disability (*n* = 70)	*F/χ^2^*	*p* value
ICOPE screening score (mean ± SD)	2.24 ± 1.84	3.49 ± 1.98	4.80 ± 2.22	4.30 ± 1.29	23.025	<0.001
Scores for specific intrinsic capacity domains (mean ± SD)						
Cognitive function	20.81 ± 7.57	18.82 ± 9.35	11.34 ± 9.58	8.01 ± 9.28	4.463	0.005
Motor function	5.91 ± 3.54	4.43 ± 3.33	0.63 ± 1.46	0.26 ± 0.99	64.578	<0.001
Vitality function	22.81 ± 3.94	20.45 ± 3.84	18.83 ± 4.36	19.07 ± 3.52	2.542	0.057
Vision function	0.93 ± 0.54	1.14 ± 0.68	1.23 ± 0.69	1.10 ± 0.59	0.124	0.946
Hearing function	5.54 ± 2.80	6.06 ± 2.95	5.00 ± 3.20	4.39 ± 3.88	1.112	0.345
Psychological function	4.46 ± 4.89	4.47 ± 5.38	5.54 ± 1.52	4.60 ± 5.14	2.130	0.098

**Figure 1:**
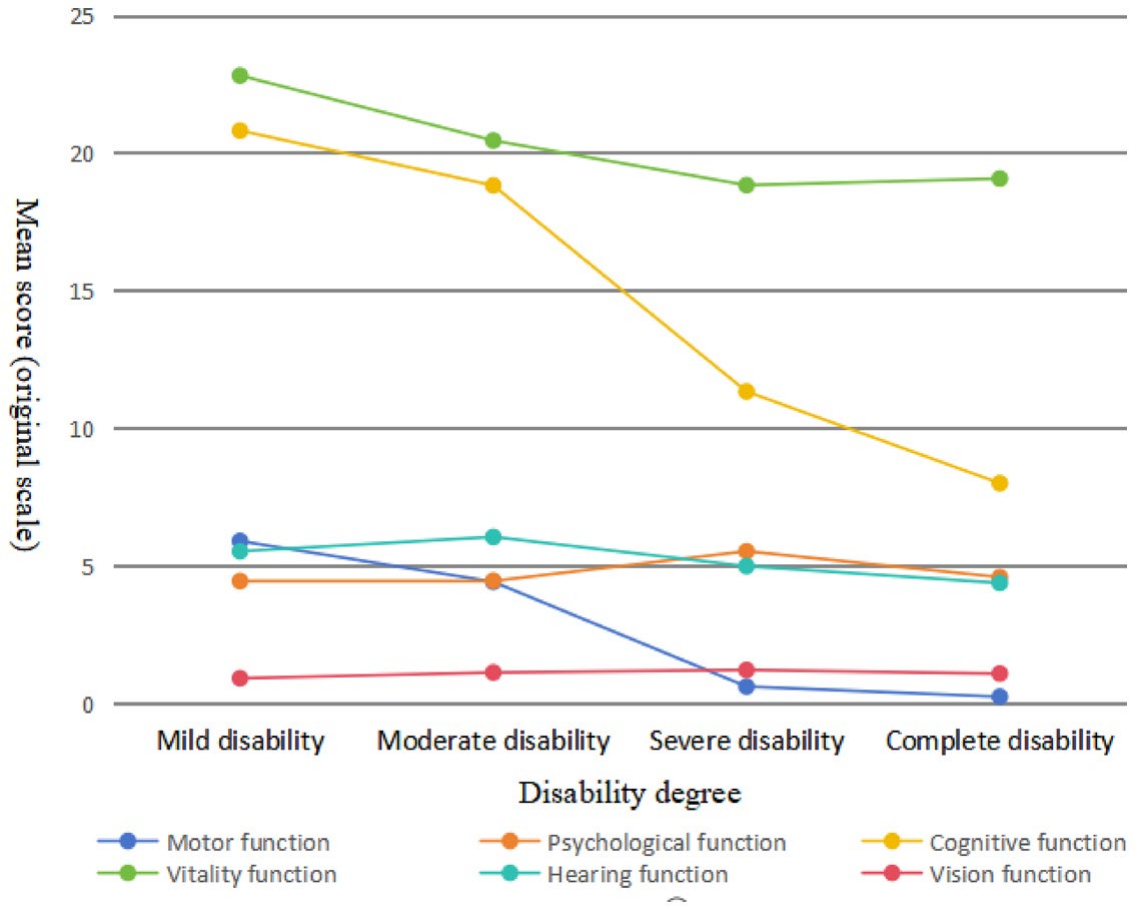
Intrinsic capacity various domains scores across disability degrees in older adults with disabilities.

The results showed differences in average scores across IC various domains among different disability degrees. Especially cognitive function average scores showed a significant difference (*F* = 4.463; *p* = 0.005). Cognitive function scores were lower in groups with greater disability degree (mild: 20.81 ± 7.57, moderate: 18.82 ± 9.35, severe: 11.34 ± 9.58, complete disability: 8.01 ± 9.28) (Table 2). Older adults with different disability degrees also showed significant differences in average motor function scores (*F* = 64.578; *p* < 0.001). Motor function scores were lower in groups with greater disability degree (mild: 5.91 ± 3.54, moderate: 4.43 ± 3.33, severe: 0.63 ± 1.46, complete disability: 0.26 ± 0.99) (Table 2). The largest difference in motor function scores was observed between the moderate and severe disability groups. Vitality and psychological scores differed across disability groups. Vision and hearing scores did not change consistently as disability degree increased.

Additionally, the impairment of IC various domains at different disability degrees in older adults with disabilities was shown in Table 3. Cognitive impairment increased with disability degree. Rates were higher in the severe (82.9%) and complete disability (85.7%) than mild (42.6%) and moderate disability (49.0%). The rate of motor function impairment increased with disability degree. The proportion of severe and complete disability reached 100%. Vitality function was more common at higher disability degree. Vision and hearing impairments varied little across disability groups and showed no clear monotonic trend. In psychological function, older adults with mild (35.2%), moderate (42.9%), and complete disability (47.1%) showed a similar trend, while the impairment proportion in severely older adults with disabilities was much higher than other three groups (80.0%).

**Table 3:** Impairment of various domains at different disability degrees in older adults (*n* = 208).

Variable	Mild disability (*n* = 54)	Moderate disability (*n* = 49)	Severe disability (*n* = 35)	Complete disability (*n* = 70)
Impairment of intrinsic capacity, *n* (%)				
Cognitive impairment	23 (42.6)	24 (49.0)	29 (82.9)	60 (85.7)
Motor function impaired motor function	42 (77.8)	45 (91.8)	35 (100.0)	70 (100.0)
Vitality function (nutritional risk and malnutrition)	32 (59.3)	40 (81.6)	30 (85.7)	66 (94.3)
Vision decline	44 (81.5)	41 (83.7)	31 (88.6)	61 (87.1)
Hearing decline	22 (40.7)	16 (32.7)	17 (48.6)	33 (47.1)
Psychological function (mild depression or higher)	19 (35.2)	21 (42.9)	28 (80.0)	33 (47.1)

Spearman’s correlation analysis analysis was used to examine the associations between disability degree and IC various domains (Table 4). Disability degree showed a significant positive correlation with the ICOPE screening score (r = 0.494, *p* < 0.01), and a significant negative correlation with motor function (r = −0.754, *p* < 0.01), cognitive function (r = −0.513, *p* < 0.01), and vitality function (r = −0.338, *p* < 0.01). Moreover, disability degree did not show a significant correlation with psychological function (r = 0.055, *p* > 0.05), hearing function (r = −0.134, *p* > 0.05), and vision function (r = 0.100, *p* > 0.05).

**Table 4:** Correlation analysis of disability degree, ICOPE screening score, and domain-specific IC measures (r values).

Variable	Disability degree	ICOPE screening score	Motor function	Psychological function	Cognitive function	Vitality function	Hearing function	Vision function
Disability degree	1.000							
ICOPE screening score	0.494**	1.000						
Motor function	−0.754**	−0.419**	1.000					
Psychological function	0.055	0.335**	−0.160*	1.000				
Cognitive function	−0.513**	−0.388**	0.494**	−0.053	1.000			
Vitality function	−0.338**	−0.486**	0.393**	−0.142*	0.210**	1.000		
Hearing function	−0.134	−0.199**	0.228**	0.092	0.497**	0.275**	1.000	
Vision function	0.100	0.320**	−0.005	0.388**	0.021	0.046	0.135	1.000

*p < 0.05, **p < 0.01.

Used ordinal logistic regression to examine the associations between IC domains and disability degree (Table 5). The results showed that motor function (*β* = −0.421, *p* < 0.001), psychological function (*β* = −0.053, *p* = 0.008), cognitive function (*β* = −0.093, *p* < 0.001), and vitality function (*β* = −0.089, *p* = 0.025) were significantly associated with disability degree. Hearing (*β* = 0.177, *p* = 0.001) was positively associated with disability degree. Vision (*β* = 0.457, *p* = 0.055) also showed a positive coefficient but did not reach statistical significance (*p* = 0.055).

**Table 5:** Ordinal logistic regression analysis of disability degree.

Variable	β	S.E.	Wald value	*p* value	95% CI	
					Lower limit	Upper limit
Motor function	−0.421	0.058	51.92	<0.001	−0.536	−0.307
Psychological function	−0.053	0.020	7.110	0.008	−0.091	−0.014
Cognitive function	−0.093	0.018	25.460	<0.001	−0.129	−0.057
Vitality function	−0.089	0.040	5.055	0.025	−0.167	−0.011
Hearing function	0.177	0.054	10.775	0.001	0.071	0.283
Vision function	0.457	0.238	3.688	0.055	−0.009	0.923

S.E. = Standard Error, 95% CI = 95% Confidence Interval.

## 4 Discussion

This study found that among older adults with disabilities in nursing institutions, disability degree showed a significant association with IC. However, different IC domains showed varied performance. As disability degree increased, motor and cognitive functions continued to decline. Vitality function scores did not further decline in the complete-disability group, while the proportion of vitality function continued to increase. Vision and hearing functions changed relatively little. Psychological impairment was highest in the severe disability group but was lower in the complete disability group. These findings indicated that after disability occurred, different IC domains did not decline synchronously as disability degree increased. Previous studies mainly examined IC decline as a predictor of later disability onset (16, 18). From this view, IC was not limited to the period before disability and could still provide functional information after disability occurred. It also identified which functions were impaired and which were preserved. Preserved capacity could be important for maintaining daily function and limiting further care dependence. Therefore, the findings suggested that long-term care should not rely on disability level alone. IC assessment could help identify the functional status of each older adult with disabilities and the areas that needed more support, allowing more targeted care.

Among the IC domains, motor and cognitive functions showed associations with disability degree. This was consistent with their important roles in daily activities and independent living (29). Motor function in IC reflected standing balance, walking, and chair stands. Cognitive function in IC included orientation, memory, attention, recall, and language ability. Impairment in either function was associated with greater disability and dependence in daily life (30). Previous studies also showed that disability among older adults with disabilities in nursing institutions was not limited to self-care, but also involved motor function and sensory perception (31), mental and cognitive status (32), and social participation (33). This finding could be explained by the similarity between disability assessment and intrinsic capacity assessment in certain functional domains. The disability assessment used in this study, GB/T 42195-2022, also included basic motor function and mental status. These partly corresponded to motor and cognitive functions in IC (34). Although motor and cognitive assessments partly overlapped with the disability assessment, this overlap should be considered when interpreting the relationship between disability and IC. Nevertheless, motor and cognitive function remained important for daily activities and independent living. Monitoring these domains could help identify older adults who needed further assessment and support. It could also guide rehabilitation, cognitive support, and care planning in long-term care facilities.

Unlike motor and cognitive function, other IC domains did not change in the same way as disability became more severe. The proportion of impaired vitality increased with disability severity. However, vitality scores changed little between severe and complete disability. Visual impairment was common in all groups, while hearing and psychological functions varied across groups. Greater disability did not mean that all IC functions declined at the same rate or in equal proportions. Even with severe disability, some IC domains also exhibited distinct states of preservation and impairment (35). The total ICOPE screening score did not continue to increase in complete disability. This suggested that multidomainal IC did not show a simple stepwise change with increasing disability degree (36). Psychological and hearing functions showed different results in the correlation analysis and multivariable model. This suggested that when multiple IC domains were simultaneously included in the analysis, the relationships between certain domains and the disability degree could changed. Overall, disability degree reflected overall disability severity. IC could further show the specific status of different functional domains. Therefore, after disability degree was identified, IC various domain further provided information on which specific functions were impaired and preserved. These findings suggested that greater disability did not mean that all functions declined together. Some functions were already impaired in mild disability, while others remained partly preserved even in severe or complete disability. Care based only on disability level might have missed nutritional, sensory, or psychological problems in older adults with mild disability. It might also have overlooked preserved abilities in those with severe or complete disability. Assessing different IC domains could help identify functions that needed more support and help maintain function and reduce further decline and care dependence.

These multidomainal functional differences should also be understood in the nursing institutions. Previous studies showed that functional changes in older adults living in nursing institutions were influenced not only by individual factors but also by institutional and caregiving environment-related factors (36, 37). The WHO healthy aging framework stated that functional ability not only depended on IC but also on the individual’s environment and the interaction between these two factors (38). From a public health perspective, this meant that healthy aging depends not only on individual capacity but also on the care and environmental conditions in which older adults with disabilities lived. The longitudinal study by Govaerts et al. also found that adequate care and social factors were related to later functional ability. Some factors also interacted with IC domains (39). This study mainly examined the relationship between individual IC and disability degree. It did not include institutional factors such as staffing, rehabilitation resources, accessible facilities, social participation, length of stay, or care models. These findings suggested that the same IC level did not always indicate the same functional status in different care settings. Long-term care needed to consider both individual impairments and institutional factors. These factors might increase or reduce problems in daily function. Future studies needed to examine whether staffing, rehabilitation, social participation, accessible facilities, and care models affected the link between IC and functional ability. Future studies also needed to examine their links with care dependence and quality of life.

The relationship between IC and disability should also be considered over time. Longitudinal systematic reviews and meta-analyses found that lower IC and IC decline were associated with later functional decline and disability (16, 18). In the cognitive domain, Hung et al. followed participants for 6 years. They found a bidirectional relationship between cognitive function and disability. Disability showed a stronger association with later cognitive decline (40). In other words, IC could decline before disability occurred, and some functions could continue to change after disability occurred (41). Current evidence suggested a bidirectional relationship between IC and functional ability. However, this relationship was not the same across different functional outcomes. Therefore, it could not be concluded that disability and overall IC had a stable bidirectional relationship (42). This study used a cross-sectional design and only showed IC differences among older adults with different disability degrees at one time point. Therefore, disability degree alone could not fully reflect the long-term functional needs of older adults with disabilities. Attention to changes in different IC domains could provide additional information for maintaining functional ability and supporting healthy aging. This could help better understand the functional status and long-term care needs of older adults with disabilities (38). Therefore, in long-term care, disability level alone was not enough to show later functional changes. Repeated IC assessment could help detect these changes and identify older adults who needed changes in care. Future studies were needed to examine changes in different IC domains after disability and their links with care dependence, further functional decline, and quality of life, and whether targeted interventions based on these domains could improve these outcomes. This could help decide how often IC should be assessed and when more support was needed.

Taken together, IC assessment could help care move beyond disability levels and focus more on remaining function and health. Maintaining functional capacity could help older adults stay more independent, need less care, and have a better quality of life. These findings also showed the public health value of IC assessment for healthy aging.

One strength of this study was extending IC research to older adults in nursing institution who have already experienced disability. Previous IC studies mainly focused on older adults in general and on whether IC decline was linked to disability or other adverse outcomes. This study focused on older adults who already had different levels of disability and examined whether IC could still reflect their function and health status. The second strength of this study was the use of the Chinese national standard GB/T 42195-2022 to classify older adults with disabilities into levels 1–4 by disability degree. This allowed us to compare IC patterns across different disability levels and examine functional differences within the disabled population. The findings provide preliminary evidence and could inform future public health and long-term care research on older adults with disabilities in other settings.

First, the cross-sectional design could not show temporal sequence or causal direction between disability degree and IC, nor could it rule out reverse causality. Therefore, the results could not be used to decide intervention priority order or disability progression. Second, disability assessment and some IC domains assessed similar aspects of function, especially motor and cognitive function. This similar could have affected results interpretation. We did not have inter-rater reliability data. Disability degree was recorded at admission, while IC was assessed during the study. The exact time gap was unknown, so the two assessments could not have fully matched. We did not perform a sensitivity analysis because the detailed disability scores needed to recalculate disability degree were unavailable. Therefore, these results should be interpreted with caution. Third, the model did not include some possible confounders or institution-level clustering. We also did not have complete diagnostic information for the ordinal logistic regression. This could have affected the robustness of the results. Finally, the ICOPE screening tool (ranging from 0 to 9) reflected the number of positive screening items. Its measurement properties need further study. The validity of the mobile hearing test also needs further evaluation. Some assessments could also have had floor or ceiling effects. Quality of life and intervention effects were not directly assessed. Further studies are needed to explore these public health implications.

## Data Availability

The data that support the findings of this study are available from the corresponding author upon reasonable request. The data are not publicly available due to ethical restrictions and the need to protect participant privacy.
