# Supplementary material for: The relationship between disability and intrinsic capacity among older adults with disabilities in nursing institutions: a cross-sectional study
Source: Front Public Health. 2026 Oct 6;14:1900440. doi: 10.3389/fpubh.2026.1900440 (PMC13680197; doi:10.3389/fpubh.2026.1900440)
Supplement: Supplementary file 1 [file Table_1.DOCX]

**Supplementary Table S1**

**GB/T 42195—2022《Specification for ability assessment of older adults》**

**Scoring Criteria for the 26 Assessment Items and Rules for Disability Level Classification and Adjustment**

All English content presented in Supplementary Table S1 was translated by the authors from the original Chinese version of GB/T 42195—2022 and does not constitute an official English translation of the standard.

**Part A. Scoring Criteria for the 26 Assessment Items**

| Assessment Domain  (Total 90 score) | No. | Assessment Item | Item Description | Scoring Criteria and Notes |
| --- | --- | --- | --- | --- |
| Self-care ability  (0-32 score) | 1 | Eating | Ability to bring food to the mouth using appropriate utensils and swallow. | **Score 4:** Independently uses utensils to bring food to the mouth and swallow, without choking.  **Score 3:** Completes eating with guidance or reminders from others, or independently uses assistive devices, without choking.  **Score 2:** Requires minor hands-on assistance during eating; occasional choking occurs (at least once per month).  **Score 1:** Requires substantial hands-on assistance during eating; frequent choking occurs (at least once per week).  **Score 0:** Completely dependent on others for feeding, or has swallowing difficulty, or has an indwelling feeding tube. |
|  | 2 | Personal Grooming | Includes washing face, brushing teeth, combing hair, shaving, trimming fingernails and toenails, etc. | **Score 4:** Completes all grooming activities independently.  **Score 3:** Completes the activity with guidance or reminders from others.  **Score 2:** Requires assistance from others, but completes the activity mainly by oneself.  **Score 1:** Primarily relies on others but can cooperate.  **Score 0:** Completely dependent on others and unable to cooperate. |
|  | 3 | Bathing | Ability to wash and dry the body. | **Score 4:** Bathes independently without assistance.  **Score 3:** Completes the activity with guidance or reminders from others.  **Score 2:** Requires assistance from others, but completes the activity mainly by oneself.  **Score 1:** Primarily dependent on others but able to cooperate.  **Score 0:** Completely dependent on others and unable to cooperate. |
|  | 4 | Dressing and Undressing (Upper Body) | Putting on or taking off upper-body clothing, fastening buttons, zipping up, etc. | **Score 4:** Completes the activity independently without assistance.  **Score 3:** Completes the activity with guidance or reminders from others.  **Score 2:** Needs assistance from others. The older adult performs the main part of the activity.  **Score 1:** Primarily dependent on others but able to cooperate.  **Score 0:** Completely dependent on others and unable to cooperate. |
|  | 5 | Dressing and Undressing (Lower Body and Footwear) | Putting on/off pants, shoes, socks, etc. | **Score 4:** Completes the activity independently without assistance.  **Score 3:** Completes the activity with guidance or reminders from others.  **Score 2:** Requires assistance from others, but completes the activity mainly by oneself.  **Score 1:** Primarily dependent on others but able to cooperate.  **Score 0:** Completely dependent on others and unable to cooperate. |
|  | 6 | Urinary Continence | The ability to control and void urine. | **Score 4:** Can control urination independently; both voiding frequency and urinary control are normal.  **Score 3:** Can independently control urination frequency during the day. Urination is more frequent at night, and urinary control is poor. Alternatively, the older adult independently uses diapers, absorbent pads, or similar aids.  **Score 2:** Can independently control urination during most of the day. Occasional urinary incontinence occurs (<1 time/day but >1 time/week). Urinary control is poor at night. Alternatively, the older adult needs a small amount of assistance to use diapers, absorbent pads, or similar aids.  **Score 1:** Cannot control urination during most of the day (≥1 time/day, but control is not completely lost). Urinary incontinence occurs at night. Alternatively, the older adult needs substantial assistance to use diapers, absorbent pads, or similar aids.  **Score 0:** Urinary incontinence with complete inability to control urination, or use of an indwelling urinary catheter. |
|  | 7 | Bowel Continence | The ability to control and expel feces. | **Score 4:** Normal independent control of defecation.  **Score 3:** Constipation or fecal incontinence occurs occasionally (<1 time/week). Alternatively, the older adult independently uses a glycerin enema, absorbent pads, or similar aids.  **Score 2:** Constipation or fecal incontinence occurs frequently (<1 time/day but >1 time/week). Alternatively, the older adult needs a small amount of assistance to use a glycerin enema, absorbent pads, or similar aids.  **Score 1:** Constipation or fecal incontinence occurs most of the time (≥1 time/day), but control is not completely lost. Alternatively, the older adult needs substantial assistance to use a glycerin enema, absorbent pads, or similar aids.  **Score 0:** Has severe constipation or complete fecal incontinence. The older adult needs assistance from others for defecation or skin cleaning. |
|  | 8 | Toileting | Uses the toilet for urination or defecation and cleans the body.  Note: The assessment emphasizes unfastening the trousers before toileting. After toileting, the older adult cleans the body and puts the trousers back on. | **Score 4:** Completes the activity independently without assistance.  **Score 3:** Completes the activity with guidance or reminders from others.  **Score 2:** Requires assistance from others, but completes the activity mainly by oneself.  **Score 1:** Primarily dependent on others but able to cooperate.  **Score 0:** Completely dependent on others and unable to cooperate. |
| Basic motor ability  (0-15 score) | 9 | Bed Mobility | Turning over in bed, sitting up, and lying down. | **Score 4:** Completes the activity independently without assistance.  **Score 3:** Completes the activity with guidance or reminders from others.  **Score 2:** Requires assistance from others, but completes the activity mainly by oneself.  **Score 1:** Primarily dependent on others but able to cooperate.  **Score 0:** Completely dependent on others and unable to cooperate. |
|  | 10 | Bed–Chair Transfer | The transition process from sitting to standing, and then from standing back to sitting. | **Score 4:** Completes the activity independently without assistance.  **Score 3:** Completes the activity with guidance or reminders from others.  **Score 2:** Requires assistance from others, but completes the activity mainly by oneself.  **Score 1:** Primarily dependent on others but able to cooperate.  **Score 0:** Completely dependent on others and unable to cooperate. |
|  | 11 | Walking on Level Ground | Moves on the ground by alternating the feet. One foot is always in front of the other.  Note: This includes walking with assistance from another person and walking with assistive devices. | **Score 4:** Independently walks approximately 50 m without assistance and without fall risk.  **Score 3:** Can walk approximately 50 m on level ground. There is a risk of falling. The older adult needs supervision or guidance from another person, or uses crutches, a walker, or similar assistive devices.  **Score 2:** Requires minor physical assistance while walking.  **Score 1:** Requires substantial physical assistance while walking.  **Score 0:** Unable to walk. |
|  | 12 | Stair Climbing | Ascends and descends stairs continuously by alternating the feet. | **Score 3:** Can independently go up and down 10–15 consecutive steps. No assistance is needed.  **Score 2:** Completes the activity with guidance or reminders from others.  **Score 1:** Requires assistance from others, but completes the activity mainly by oneself.  **Score 0:** Primarily relies on assistance from others but is able to cooperate; or is completely dependent on assistance from others and unable to cooperate. |
| Mental status  (0-28 score) | 13 | Time Orientation | The ability to know and confirm time. | **Score 4:** Clear orientation to year and month; the date (or day of the week) may differ by one day.  **Score 3:** The sense of time has somewhat declined; the ability to distinguish between years, months, days (or the day of the week) is impaired (with a difference of two or more days).  **Score 2:** Poor time orientation; year, month, and date are unclear, but the person can identify the first or second half of the year or the season.  **Score 1:** Very poor time orientation; year, month, and date are unclear, but the person can distinguish morning from afternoon or daytime from nighttime.  **Score 0:** No sense of time. |
|  | 14 | Spatial Orientation | The ability to know and identify one's location. | **Score 4:** Can go out alone within the usual living area. For example, the older adult can shop alone within the residential community.  **Score 3:** Cannot go out alone. Accurately knows the address of the usual place of daily living.  **Score 2:** Cannot go out alone. Knows considerable address information related to the place of daily living.  **Score 1:** Cannot go out alone. Knows little address information about the place of residence or daily living.  **Score 0:** Cannot go out alone and has no spatial orientation. |
|  | 15 | Person Orientation | The ability to know and identify people. | **Score 4:** Can recognize people who have lived with the older adult for a long time. Can address them and knows the relationship.  **Score 3:** Can recognize most people living together; can address or identify relationships with them.  **Score 2:** Can recognize some family members or caregivers who live with them daily; can address or identify their relationships.  **Score 1:** Only recognizes oneself or very few family members or caregivers who live with the older adult.  **Score 0:** Recognizes no one, including self. |
|  | 16 | Memory | Short-term, recent, and remote memory abilities. | **Score 4:** Maintains memory function adapted to social demands and age, with the ability to recall information in its entirety.  **Score 3:** Mild memory disturbance or inability to recall (unable to recall immediate information; after 5 minutes, only 0–1 of 3 words can be recalled).  **Score 2:** Symptoms of moderate memory impairment or impaired recall (unable to recall recent events, such as not remembering what was eaten at the previous meal).  **Score 1:** Severe memory disturbance or inability to recall. Remote memories cannot be recalled, such as not remembering old friends.  **Score 0:** Complete memory disorganization or complete inability to correctly recall past events. |
|  | 17 | Comprehension | The ability to understand both verbal and non-verbal information (which can be aided by the regular use of hearing aids or other assistive devices), namely, the ability to understand what others are saying. | **Score 4:** Understands spoken language normally.  **Score 3:** Can understand what others say, but requires additional time.  **Score 2:** Has difficulty understanding and requires frequent repetition or simplified verbal expression.  **Score 1:** Has severe difficulty understanding. Needs substantial help from others.  **Score 0:** Completely unable to understand what others say. |
|  | 18 | Expression | The ability to express information, including both oral and non-oral forms—namely, expressing one's own thoughts. | **Score 4:** Can express their thoughts normally.  **Score 3:** Can express own needs, but requires additional time.  **Score 2:** Has difficulty expressing needs. Frequent repetition or simplified verbal expression is required.  **Score 1:** Severe expressive difficulty requiring substantial assistance.  **Score 0:** Completely unable to express needs. |
|  | 19 | Aggressive Behavior | Physical aggression (such as hitting, kicking, pushing, biting, grabbing, or throwing objects) and verbal aggression (such as cursing, verbal threats, or screaming).  Note: Long-term behavioral status. | **Score 1:** No aggressive behavior.  **Score 0:** Aggressive behavior occurred within the past month. |
|  | 20 | Depressive Symptoms | Symptoms may include low mood, loss of interest, reduced energy levels, as well as the emergence of delusions, hallucinations, suicidal ideation, or suicidal behavior.  Note: Prolonged negative emotions. | **Score 1:** No depressive symptoms are present.  **Score 0:** Negative emotions were present within the past month. |
|  | 21 | Level of Consciousness | The extent of an organism's ability to respond to stimuli from both itself and its surrounding environment, encompassing both conscious and sustained states of arousal.  Note: An older adult in a coma is directly classified as having severe disability. | **Score 2:** The patient is conscious and can respond appropriately to their surroundings.  **Score 1:** Somnolence manifests as an excessively prolonged sleep state. The elderly patient can be aroused by calling or by moving their limbs; they may then engage in appropriate conversation or follow instructions, and will resume sleep once the stimulation is ceased; other symptoms include confusion of consciousness, distractibility, inability to perceive external stimuli clearly, impaired spatial and temporal orientation, slowed comprehension, and blurred or disjointed memory.  **Score 0:** Stupor: under normal circumstances, general external stimuli cannot induce awakening; however, exposure to relatively strong stimuli may result in a brief period of conscious awareness, during which the patient can provide brief responses to questions; once the stimulus is removed, the patient quickly returns to a sleep state; or comatose state: characterized by loss of consciousness, loss of voluntary movement, and complete absence of response to any general stimuli. |
| Sensory perception and social participation  (0-15 score) | 22 | Vision | The ability to perceive light and the size and shape of objects; assessment should be conducted at the individual's best corrected visual acuity. | **Score 2:** Normal visual acuity.  **Score 1:** Can read large fonts clearly, but cannot read standard typefaces in books or newspapers; vision is limited—cannot see large headlines in newspapers, but can still recognize objects.  **Score 0:** Can only see light, color, and shape; or is completely blind. |
|  | 23 | Hearing | The ability to distinguish the direction of sound, pitch, volume, and timbre (which can be aided by the use of hearing aids or other assistive devices). | **Score 2:** Normal hearing.  **Score 1:** Unable to hear clearly when the speaker speaks softly or when the speaker is more than 2 m away; normal communication is somewhat difficult, requiring a quiet environment or louder speech to be heard.  **Score 0:** Can hear only part of what is said when the speaker speaks loudly or very slowly, or is completely deaf. |
|  | 24 | Managing Daily Affairs | Plan, arrange, and complete daily tasks, including but not limited to doing laundry, making small purchases, and managing medication. | **Score 4:** Capable of independently planning, arranging, and completing daily tasks without any assistance.  **Score 3:** When planning, arranging, or completing daily tasks, assistance from others is required for supervision or guidance.  **Score 2:** Requires minimal assistance when planning, arranging, and completing daily tasks.  **Score 1:** Significant assistance is required when planning, arranging, and completing daily tasks.  **Score 0:** Completely relying on others for daily tasks. |
|  | 25 | Using Transportation for Outings | — | **Score 3:** Can go out on their own by riding a bicycle or using public transportation.  **Score 2:** Can take a taxi on their own but cannot use other public transportation to go out.  **Score 1:** Can use public transportation when assisted or accompanied by another person.  **Score 0:** Can only take a taxi or use a private car to go out with the assistance of others; cannot go out at all, or must rely entirely on assistance when going out. |
|  | 26 | Social Interaction Ability | — | **Score 4:** Engages with society, demonstrates a certain ability to adapt to the social environment, and interacts with others appropriately.  **Score 3:** Can adapt to simple environments and actively engage with others; at first meeting, it may be difficult for others to detect any intellectual difficulties, and the older adult cannot understand metaphors.  **Score 2:** Is socially withdrawn. Can interact passively but does not actively interact with others. Uses many inappropriate words or phrases in conversation. Is easily deceived.  **Score 1:** Can barely interact with others; the content of speech is unclear, and facial expressions are inappropriate.  **Score 0:** Unable to interact with others. |

Note: An em dash (—) indicates that no separate item description is provided in GB/T 42195—2022.

**Part B. Classification of Disability Levels**

| **Disability Level** | **Level Name** | **Total Score** |
| --- | --- | --- |
| 0 | Intact ability | 90 |
| 1 | Mildly impaired ability (mild disability) | 66～89 |
| 2 | Moderately impaired ability (moderate disability) | 46～65 |
| 3 | Severely impaired ability (severe disability) | 30～45 |
| 4 | Complete loss of ability (complete disability) | 0～29 |

**Part C. Rules for Disability Level Adjustment**

| **Circumstance** | **Adjustment Rule** |
| --- | --- |
| In a comatose state | Directly classified as complete loss of ability (complete disability). If the state of consciousness changes, reassessment shall be conducted. |
| Confirmed diagnosis of dementia (F00–F03) | The original ability level shall be increased by one level. |
| Other mental and behavioral disorders (F04–F99) diagnosed by a psychiatrist | The original ability level shall be increased by one level. |
| Two or more care-related risk events within the past 30 days (e.g., falls, choking, suicide, self-injury, getting lost, etc.) | The original ability level shall be increased by one level. |

Note: F00–F99 are diagnostic codes for mental and behavioral disorders in ICD-10 (International Classification of Diseases, 10th Revision).
